# Patterns and factors associated with healthcare utilisation in Cambodia: a cross-sectional study based on the World Health Survey Plus 2023

**DOI:** 10.1136/bmjph-2024-001416

**Published:** 2025-02-11

**Authors:** Srean Chhim, Paul Kowal, Chamnab Ngor, Sereyraksmey Long, Poppy Walton, Khin Thiri Maung, Grace Marie Ku, Kerstin Klipstein-Grobusch, Nawi Ng, Por Ir, Chhorvann Chhea, Heng Sopheab

**Affiliations:** 1 School of Public Health, National Institute of Public Health, Phnom Penh, Cambodia; 2 Julius Center for Health Sciences and Primary Care, University Medical Center Utrecht, Utrecht, The Netherlands; 3 National Centre for Epidemiology and Population Health, Australian National University, Canberra, Australian Capital Territory, Australia; 4 HelpAge International, London, UK; 5 School of Public Health and Community Medicine, University of Gothenburg, Gothenburg, Sweden; 6 Institute of Tropical Medicine, Antwerp, Belgium; 7 Department of Gerontology, Vrije Universiteit Brussel, Brussel, Belgium; 8 Department of Preventive Medicine, University of Santo Tomas, Manila, Philippines; 9 Faculty of Health Sciences University of the Witwatersrand, Johannesburg, South Africa; 10 Management team, National Institute of Public Health, Phnom Penh, Cambodia

**Keywords:** Public Health, Sociodemographic Factors, Secondary Prevention

## Abstract

**Background:**

The Cambodian government aims to boost healthcare utilisation in public facilities and reduce the spending burden for disadvantaged households. This study aims to describe patterns of public and private outpatient and inpatient healthcare use and investigate the factors associated with public healthcare usage in Cambodia.

**Method:**

A cross-sectional study was conducted in all provinces in Cambodia, including the capital, Phnom Penh. The analysis included 4603 individuals aged ≥18 who had received care within the last 12 months.

**Results:**

Around 9% of outpatient and 50% of inpatient visits were made to public healthcare facilities. The number of outpatient visits made to public healthcare compared with private healthcare facilities was significantly higher in women (aOR 1.4, 95% CI 1.1, 1.8), living in rural settings (aOR 1.4, 95% CI 1.1, 1.7), those in the poorest (aOR 1.7, 95% CI 1.2, 2.3) and poor (aOR 1.5, 95% CI 1.1, 2.1) compared with the richest wealth quintiles, and respondents with insurance coverage (aOR 2.0, 95% CI 1.6, 2.5). The number of inpatient visits made to public healthcare compared with private healthcare facilities was significantly higher in the poorest (aOR 2.4, 95% CI 1.4, 3.9), poor (aOR 2.4, 95% CI 1.5, 4.0) and middle (aOR 2.5, 95% CI 1.5, 4.1) compared with those in the richest wealth quintiles and respondents with insurance coverage (aOR 2.1, 95% CI 1.5, 3.2).

**Conclusion:**

Our study shows that private healthcare dominates outpatient services in Cambodia, while public healthcare is more significant for inpatient care. Individuals with low socioeconomic status and those with insurance showed higher public healthcare utilisation for outpatient and inpatient services, with women more likely to use public outpatient care. To progress towards universal health coverage, it is essential to improve public healthcare quality, especially in rural areas, expand service coverage and social health protection and develop strategies to engage the private sector.

**WHAT IS ALREADY KNOWN ON THIS TOPIC**

- Previous research has suggested that most outpatient and almost half of the inpatient visits were made to private healthcare providers.

**WHAT THIS STUDY ADDS**

- The use of public healthcare services for outpatient visits remains low after several years of government-supported efforts to increase its use.
- The use of public healthcare for inpatients is comparable to private healthcare services.
- The use of public healthcare for outpatient and inpatient is higher in women, poor and poorest groups, and those with health insurance entitlement.

**HOW THIS STUDY MIGHT AFFECT RESEARCH, PRACTICE OR POLICY**

- This study provides timely information to the Cambodian government for their efforts to improve and increase the use of the public healthcare system to contribute to progress towards universal health coverage.

## Introduction

 Many countries are working towards universal health coverage (UHC) with the objective of providing access to high-quality healthcare services without creating financial strain.^1^

While there is an ongoing debate on the mix of healthcare provision by public and private providers in low- and middle-income countries, most countries use both.^2^ In resource-constrained environments, private healthcare providers enhance healthcare accessibility, an essential dimension of UHC. However, users are more likely to bear health costs, which increases the risk of financial vulnerability.^2^ A reorientation of health systems towards free primary healthcare at the point of use would be ideal for public health systems working towards UHC. However, until this happens, a mix of public and private providers will be a reality.^3 4^ Estimates of private-sector care providers across WHO regions account for up to 40% of all health services in the WHO Americas, Africa and Western Pacific regions; 57% of services in the Southeast Asian region and 62% in the Eastern Mediterranean region.^5^ In Cambodia (WHO’s Western Pacific region), private healthcare has experienced significant growth since 1994, after the government decided to permit government practitioners to engage in ‘dual practice’ by working for private practices outside their regular public practice working hours.^6^ The use of public outpatient care services in the last 30 days in Cambodia was low at around 20%, whereas similar rates of public and private service usage were observed for inpatient visits (47.3% vs 43.0%) in 2014.^7^ Cumulative evidence of low public healthcare use preceded a notable initiative to improve healthcare quality and equity in the public healthcare system, the 2017 Health Equity and Quality Improvement Project (H-EQIP), with a budget of US$175 million.^8^ The H-EQIP aimed to strengthen health service delivery, improve financial protection and equity and ensure sustainable and responsive health systems.^8^ Its first phase ended in 2020, and the second phase began in 2021 for another 5 years with a budget of US$299 million.^8 9^ These efforts aim to boost healthcare utilisation in public facilities and reduce the spending burden for disadvantaged households.

The current study has two main objectives. First, it described the use of public and private healthcare for outpatient and inpatient care in Cambodia. Second, we aimed to determine factors associated with public healthcare usage.

### Context

#### Cambodian health system structure

Cambodia’s health system is pluralistic, with services provided in public healthcare facilities and a large private sector.^6^ Health system governance is organised into three tiers: national or central level, provincial level and operational district (OD) level.^6^ In 2019, the Cambodian government decentralised health service delivery, human resources and financing management to the provincial administration, resulting in slight changes to the roles at central and provincial levels.^10^ The central level now focuses on policy development and monitoring policy implementation, while the provincial level has gained greater authority in organising and managing health service delivery. At the OD level, which functions as the primary healthcare system, healthcare is delivered locally under the umbrella of provincial health departments.^6^ Each OD has at least one district referral hospital, with a few ODs having two district hospitals. Each OD has a population of 100 000–200 000.^6^

#### Public healthcare

The public healthcare system dominates inpatient care and preventive services, such as reproductive, maternal, neonatal and child health and vaccinations.^6^ As^11^ of 2022, there were 1548 public healthcare facilities across Cambodia.^11^ These were composed of four levels of facilities: 12 national hospitals and centres, 132 referral hospitals (provincial or district level), 1288 health centres and 125 health posts.^11^ The health centres are entry points of care providing basic treatment, health promotion and preventive services. Health posts are available in remote areas with small populations and offer similar services to health centres but on a smaller scale.^6^ District and provincial referral hospitals accept patients who self-refer or have been referred by health centres. They provide general services, including treatment and surgery. National hospitals have advanced services, including specialised services.^6^

#### Private healthcare

Private healthcare is generally more easily accessible for many Cambodians. The private sector includes cabinets (medical consultation rooms with up to two medical beds), pharmacies, clinics, polyclinics and hospitals.^6 12^ As of 2022, 16 185 private healthcare facilities and 3381 pharmacies and sub-pharmacies were operating in Cambodia. Approximately 93% of the facilities were cabinets offering medical consultation services. The second most common facilities (approximately 6%) were clinics and polyclinics, with between 10 and 49 beds, offering specialty, laboratory and radiology services.^611^,^13^ Over 50% of healthcare workers in private facilities are government personnel practising in the public sector.^6 14^

#### Healthcare expenditure and social health insurance

Out-of-pocket expenditure (OOPE) is the primary source of funding for healthcare services, covering about 55% of national health expenditure in 2021, while government expenses for health remain low at around 26.6% (7.5% of GDP in 2021), with another portion coming from external funding. Many people in Cambodia continue to purchase medications from pharmacies or drug stores for self-treatment without a doctor’s prescription.^6 12 13^ The annual OOPE per capita was US$67 (with an annual household income of less than US$1600) or approximately 4.1% of the GDP in 2021.^15^,^17^

To reduce the health cost burden on users, the government introduced two main social protection schemes: the Health Equity Fund (HEF) and the National Social Security Fund (NSSF).^18^,^21^ HEF was established in 2000 to provide exemptions for public healthcare facilities in low-income households. As of December 2023, HEF covered over 3 million people.^20^ Starting its implementation in 2008, NSSF has covered private formal employees and civil servants. As of December 2024, the NSSF has covered 3.12 million individuals.^21^ Recently, the NSSF has taken a significant step towards achieving universal insurance coverage by introducing a health insurance scheme for self-employed individuals and their dependents. By December 2023, almost 90 000 people had been enrolled in the new scheme.^22^ The HEF and NSSF cover approximately 39% of the population, indicating that a significant portion remains without social health protection.

### Conceptual framework

The decision to choose between public and private healthcare can be influenced by numerous factors.^23 24^ Several studies have been conducted to identify the factors associated with public healthcare usage in Cambodia. However, the target population has varied across these studies, and one study found that older individuals with chronic conditions, having social protection entitlement (HEF or NSSF entitlement) and low socioeconomic status are independently associated with a higher use of public healthcare.^25^ Additionally, in 2021, approximately 90% of antenatal care (ANC) took place in public healthcare facilities, resulting from a long history of improving complete maternal and childcare packages to reduce maternity and child mortality rates. This suggests that the sex of participants may influence the choice between the public and private healthcare sectors because of the different needs of the services.^26^

Similar findings were observed in a study from India, which found that the low wealth quintile and low educational level were the main factors influencing the choice to use public health services.^23^

Generally, at the systemic level, people avoid public healthcare primarily because of unfriendliness and long waiting hours.^27 28^ A study in Cambodia^27^ and a recent study in Vietnam^28^ suggested that long waiting times and social factors, such as word of mouth, staff attitudes and marketing, significantly impact people’s choice of private healthcare over public healthcare. In summary, individual-level factors, such as sex, age and education, influence healthcare usage choices due to different service needs (online supplemental figure 1). Socioeconomic class, measured as a wealth quintile, plays a crucial role because it determines the ability to pay, pushing some individuals to seek affordable healthcare or where they can receive it for free (online supplemental figure 1). Private-sector services have a larger market in Cambodia’s urban areas and other low-resource settings; therefore, residency can also be a potential factor influencing choice (online supplemental figure 1). We included these factors in our analysis, considering Cambodia’s unique context.

## Materials and methods

### Data source

Our study used primary data from the 2023 World Health Survey Plus (WHS+) conducted in Cambodia.^29^ This survey was a collaborative effort between the National Institute of Public Health, WHO, HelpAge International and the University of Gothenburg. The WHS+ is a WHO initiative to monitor progress in health-related sustainable development goals and UHC in low- and middle-income countries, including Cambodia. The original protocol of the WHS+ is shown in online supplemental file 1. The study included adults aged ≥18 years residing in selected households and who provided informed consent (online supplemental figure 2). Only one person per household was interviewed. Out of 6154 eligible households, 5271 individual questionnaires were completed, resulting in a response rate of 85.6%. All respondents were asked questions about healthcare utilisation. However, only those who reported receiving healthcare in the 12 months before the interview were included in the final analytical sample, leaving 4784 individuals.

### Sampling and recruitment process

The sampling was nationally representative. A three-stage cluster sampling was performed. For the primary sampling unit, 276 villages out of 14 568 were selected using proportional probability sampling. The National Institute of Statistics (NIS) has provided the most recent village list with the size of households and residents in Cambodia as of 2021.

For the secondary sampling unit, we randomly chose 22 households per village, and this process involved three stages. First, we created a sampling frame for the physical structure of each village using high-resolution satellite images and GIS software. The geo-coordinates of all buildings in each village were marked in the Google Earth software. We extracted a line-listing CSV file from this and generated the sampling frame. To obtain 22 eligible households for the interviews, we selected 44 buildings per village. The reason for selecting 44 points was from the pre-testing, which showed that 50% of the buildings were eligible households. The eligible households consisted of private households with at least one permanent member aged 18 years or older who had resided in the selected villages for a minimum of 6 months within the past year. For the tertiary sampling unit, we interviewed one eligible individual for the individual questionnaire and one household head for the household questionnaire in each household. After all household members were listed on the computer-assisted programme interview (CAPI), the CAPI randomly selected one person aged 18 years or older. The person was not replaced if he/she could not participate for any reason.

### Data collection

The WHS+ Cambodia was implemented between 12 March and 31 May 2023. Fourteen teams comprised 14 team leaders, 56 interviewers and 14 biomarker collectors. Each survey team underwent a 10-day training programme, which included 6 days of survey technique and tool training, 2 days dedicated to biomarkers and performance tests, 1 day for pilot testing and 1 day for debriefing and troubleshooting.

The survey included nine modules that covered a wide range of topics, including sociodemographic characteristics, chronic conditions and inpatient and outpatient healthcare utilisation.

### Ethics and oversight

The WHS+ Cambodia protocol was approved by the National Ethics Committee for Health Research (NECHR), dated 20 July 2022 (approval code: No. 221 NECHR). Initially, the study protocol required written consent from participants. Due to respondents’ concerns about the potential misuse of signatures on written consent forms, we sought approval from the NECHR to switch from written to verbal consent for our research.

### Patients and public involvement

No respondents were involved in developing the research questions, outcome measures, study design or study recruitment. We do not plan to disseminate the findings to the study respondents.

### Measures

This study focused on the most recent visits to a public or private healthcare facility. Public healthcare facilities are those managed by the government that offers medical services, such as national hospitals, provincial referral hospitals, district referral hospitals, health centres and health posts. Similar to health centres, health posts are rare and found only in remote areas. They are considered to be a lower branch of health centres; therefore, we grouped these facilities together for analysis. Private healthcare services are provided by the non-government sector, which offers medical services such as private hospitals, private clinics and polyclinics, cabinets, pharmacies, homes of trained health workers (in private hours) and visits of health workers to patients’ homes (in private hours). This category does not include traditional healing/medicine or informal allopathic providers—those without medical, nursing, midwifery or pharmacy qualifications. Healthcare services obtained outside Cambodia were also excluded from the analyses.

Our study focused on identifying the relationship between the use of public healthcare services and patient attributes, such as the location of residence (urban or rural), sex (male or female), age (18–29, 30–39, 40–49 or 50+years), level of education (none, primary, secondary, high school or higher), social protection status (insured or not insured) and wealth quintile (ranging from poorest to wealthiest). Urban or rural villages were classified according to the NIS. Please refer to online supplemental annexure 1, which provides a detailed explanation of the wealth quintile calculation.

### Analysis

Descriptive and inferential analyses were performed. Considering the age-sex and community-type population structure, post-stratification weights were used for descriptive and bivariate analyses.

We used descriptive analysis to provide statistics, using the proportion for categorical variables and median and IQR for continuous variables. For bivariate analysis, we used the X^2^ or Fisher’s exact test to identify potential categorical variables associated with higher usage of public out-patient and in-patient healthcare. All variables with a *p* value of <0.2 in bivariate analysis were included in the multivariable analysis. Generalised linear modelling with binomial specification was used in multivariable analysis. We used the stepwise method to identify the best model. The model with the smallest Akaike information criterion was selected as the final model. R programming’s gt_summary and MASS packages were used as statistical tools for this analysis.^30 31^ A two-sided p value of <0.05 was considered statistically significant in the regression analyses.

## Results

### Characteristics of participants

The analytical sample consisted of 4784 individuals who reported having received or accessed healthcare in the last 12 months, women (54.1%) and those living in rural areas (56.3%) (table 1). The participants’ median age was 38 years (IQR: 28–53), and only 15.3% had health insurance (table 1).

**Table 1:** Characteristics of World Health Survey Plus participants aged 18+ years in Cambodia

Characteristic	Unweighted	Weighted
	n=4784	n=4784
Type of community		
Urban	1901 (39.7)	43.7
Rural	2883 (60.3)	56.3
Sex of participant		
Male	1413 (29.5)	45.9
Female	3371 (70.5)	54.1
Age in year (median (IQR))	47	38 (28, 53)
Age group in year		
18–29	595 (12.4)	28.9
30–39	1012 (21.2)	24.1
40–49	980 (20.5)	17.5
50–59	985 (20.6)	13.4
60+	1212 (25.3)	16.1
Marital status		
Married or living together	3706 (77.5)	80.4
Never married	253 (5.3)	10.4
Separated/divorced/windowed	825 (17.2)	9.3
Wealth quintile		
Poorest	885 (18.5)	12.1
Poor	971 (20.3)	17.6
Middle	985 (20.6)	20.4
Rich	979 (20.5)	21.0
Richest	964 (20.2)	28.9
Educational level		
Never attended school	1059 (22.1)	14.9
Lower than primary school	1690 (35.3)	25.7
Primary school completed	1044 (21.8)	26.3
Secondary school completed	587 (12.3)	18.5
High school completed	275 (5.7)	9.6
College/pre-university/university completed	129 (2.7)	4.9
Having insurance entitlement		
Insured	832 (17.4)	15.3
Not insured	3952 (82.6)	84.7

### Healthcare utilisation

**Figure 1:**
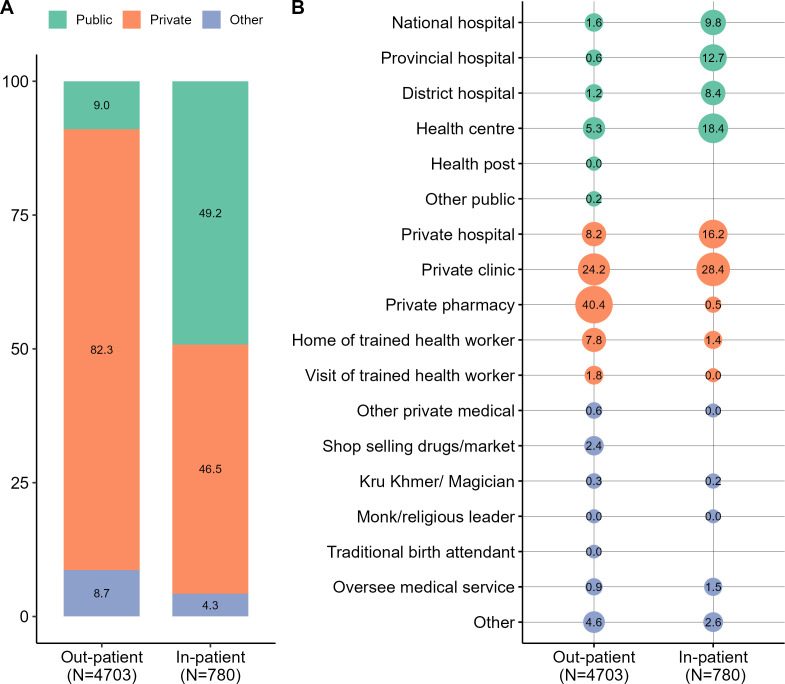
Proportions of last visits to public and private facilities in the past 12 months.

Figure 1 shows the proportion of those who reported using public and private healthcare facilities in the past 12 months. About 9.0% of outpatient and 49.2% of inpatient visits were made to public healthcare facilities. Most outpatient visits (40.4%) were to pharmacies, followed by private clinics or cabinets (24.2%), private hospitals (8.2%) and homes of trained health workers/nurses (7.8%). Public hospitals were preferred for inpatient visits (30.9%), followed by private clinics (28.4%).

### Reasons for healthcare seeking

The most common reason for outpatient visits (54.1%) was an acute condition, followed by general pain (11.6%), high blood pressure/hypertension (6.9%) and chronic pain (4.2%) (table 2).

**Table 2:** Reasons for outpatient and inpatient healthcare seeking among adults aged 18+ years in Cambodia, 2023

Characteristic	Outpatient visits			Inpatient visits		
	Overall*n=4282	Private†n=3817	Public†n=465	Overall*n=751	Private†n=381	Public†n=370
Acute conditions (diarrhoea, fever, influenza, headaches, cough, etc)	2187 (54.1)	95.6	4.4	69 (8.7)	67.1	32.9
Generalised pain (stomach, muscle or non-specific pain)	556 (11.6)	89.0	11	129 (11.7)	65.6	34.4
High blood pressure/hypertension	462 (6.9)	90.8	9.2	44 (2.7)	76.6	23.4
Chronic pain in the joints/arthritis (joints, back, neck)	188 (4.2)	88.9	11.1	24 (3.0)	50.8	49.2
Diabetes or diabetes-related complications	129 (2.7)	87.9	12.1	6 (0.9)	84.2	15.8
Problems with your mouth, teeth or swallowing	114 (3.3)	98.9	1.1	0 (0.0)	NA	NA
Maternal and perinatal conditions	76 (2.4)	34.9	65.1	152 (28.5)	36	64
Problems with heart, including unexplained chest pain	69 (1.6)	67.8	32.2	33 (3.6)	32	68
Injury (not occupation related)	45 (1.5)	74.4	25.6	16 (2.9)	34.2	65.8
Problems with your breathing	35 (0.7)	66.6	33.4	16 (1.0)	23.2	76.8
Communicable disease (Infections, malaria, tuberculosis, AIDS, etc)	24 (0.7)	56.6	43.4	15 (2.6)	74.9	25.1
Nutritional deficiencies	19 (0.3)	87.4	12.6	8 (0.5)	67.6	32.4
Depression or anxiety	12 (0.3)	35.2	64.8	1 (0.0)	0	100
Surgery	11 (0.3)	97.6	2.4	63 (9.0)	74.1	25.9
Cancer	9 (0.3)	47.8	52.2	1 (0.1)	100	0
Sleep problems	8 (0.1)	70.4	29.6	2 (0.1)	100	0
Occupation/work-related condition or injury	8 (0.0)	89.4	10.6	3 (0.1)	72.2	27.8
Stroke/ sudden paralysis of one side of the body	5 (0.1)	52.4	47.6	2 (0.3)	0	100
Other	325 (9.1)	85.9	14.1	167 (24.5)	36.9	63.1

* Column percentages.

† Row percentages.

The primary reason for an inpatient visit was maternal and perinatal care, accounting for 28.5% of stays, followed by general pain (11.7%), surgery (9.0%) and acute conditions (8.7%).

Outpatient visits to public healthcare facilities were predominantly for maternal and perinatal conditions (65.1%), depression or anxiety (64.8%) and cancer (52.2%). Similarly, inpatient visits to public healthcare facilities were primarily for problems with breathing (76.8%), injuries (65.8%) and maternal and perinatal conditions (64.0%).

### Factors associated with higher public healthcare usage for outpatient visits

In the bivariate analysis, several factors were statistically associated with public healthcare usage for outpatient visits, including the type of community, sex, marital status, wealth quintile and health insurance. After adjusting for these variables in a multivariable analysis, four variables remained significantly associated with the outcome variable (table 3). People in rural area settings were more likely to use public healthcare (aOR 1.4, 95% CI 1.1, 1.7) than people in urban settings. Women had a higher chance of using public healthcare (aOR 1.4, 95% CI 1.1, 1.8) than men. Furthermore, individuals in the poorest wealth quintile (aOR 1.7, 95% CI 1.2, 2.3) and the poor wealth quintile (aOR, 1.5 (95% CI 1.1, 2.1) were more likely to use public healthcare. Lastly, participants with insurance coverage were more likely to use public healthcare (aOR 2.0, 95% CI 1.6, 2.5) than those without insurance coverage.

**Table 3:** Factors associated with public healthcare use among outpatient service users, 2023

Characteristic	Bivariate (n=4273)			Multivariate (n=4273)		
	Private n=38 08*	Public n=465	P value	aOR	95% CI	P value
Type of community			**0.011**			
Urban	1583 (92.4)	162 (7.6)		—	—	
Rural	2225 (88.5)	303 (11.5)		1.4	1.1, 1.7	**0.002**
Sex of participant			0.069			
Male	1165 (91.7)	111 (8.3)		—	—	
Female	2643 (88.9)	354 (11.1)		1.4	1.1, 1.8	**0.002**
Age in year (median (IQR))	39 (29, 53)	39 (27, 56)	>0.9			
Age group in year			**0.045**			
18–29	456 (89.0)	55 (11.0)				
30–39	821 (92.4)	99 (7.6)				
40–49	789 (90.5)	90 (9.5)				
50–59	787 (93.0)	84 (7.0)				
60+	955 (86.3)	137 (13.7)				
Marital status			0.6			
Married or living together	2944 (89.9)	379 (10.1)				
Never married	204 (92.4)	18 (7.6)				
Separated/divorced/windowed	660 (90.9)	68 (9.1)				
Wealth quintile			0.062			
Poorest	657 (86.4)	104 (13.6)		1.7	1.2, 2.3	**0.001**
Poor	756 (89.7)	101 (10.3)		1.5	1.1, 2.1	**0.012**
Middle	789 (90.1)	88 (9.9)		1.2	0.9, 1.6	0.325
Rich	784 (88.6)	97 (11.4)		1.3	1.0, 1.9	0.072
Richest	822 (93.3)	75 (6.7)		—	—	
Educational level			0.3			
Never attended school	820 (88.1)	104 (11.9)				
Lower than primary school	1320 (89.1)	169 (10.9)				
Primary school completed	848 (92.5)	96 (7.5)				
Secondary school completed	482 (89.6)	57 (10.4)				
High school completed	234 (92.9)	25 (7.1)				
College/pre-university/University completed	104 (86.9)	14 (13.1)				
Having insurance entitlement			**<0.001**			
Insured	615 (82.6)	123 (17.4)		2.0	1.6, 2.5	**<0.001**
Not insured	3193 (91.6)	342 (8.4)		—	—	

* n (unweighted) %(weighted); Median (Q1, Q3).

aOR, Adjusted OR.

### Factors associated with higher public healthcare usage for inpatient visits

In regard to inpatient visits (table 4), the results of the multivariable analysis revealed that individuals in the poorest wealth quintile were more likely to use public healthcare (aOR 2.4, 95% CI 1.4, 3.9), those in the poor wealth quintile (aOR 2.4, 95% CI 1.5, 4.0) and the middle wealth quintile (aOR 2.5, 95% CI 1.5, 4.1), when compared with those in the highest wealth quintile. Additionally, participants with insurance coverage were more likely to use public healthcare than those without (aOR 2.1, 95% CI 1.5, 3.2).

**Table 4:** Factors associated with public healthcare use among in-patient service users, 2023

Characteristic	Bivariate (n=751)			Multivariate (n=751)		
	Private n=381	Public n=370	P value	OR	95% CI	P value
Type of community			0.5			
Urban	140 (51.6)	137 (48.4)				
Rural	241 (46.8)	233 (53.2)				
Sex of participant			0.2			
Male	85 (54.9)	72 (45.1)				
Female	296 (45.6)	298 (54.4)				
Age in year (median (IQR))	37 (26, 51)	31 (24, 46)	0.2			
Age group in year			0.4			
18–29	63 (45.3)	90 (54.7)				
30–39	76 (44.4)	81 (55.6)				
40–49	72 (63.8)	53 (36.2)				
50–59	74 (52.6)	50 (47.4)				
60+	96 (49.9)	96 (50.1)				
Marital status			0.7			
Married or living together	298 (47.4)	309 (52.6)				
Never married	15 (55.8)	11 (44.2)				
Separated/divorced/windowed	68 (53.9)	50 (46.1)				
Wealth quintile			**<0.001**			
Poorest	68 (38.3)	90 (61.7)		2.4	1.4, 3.9	**<0.001**
Poor	67 (45.5)	81 (54.5)		2.4	1.5, 4.0	**<0.001**
Middle	78 (32.1)	97 (67.9)		2.5	1.5, 4.1	**<0.001**
Rich	91 (60.0)	61 (40.0)		1.3	0.8, 2.2	0.288
Richest	77 (71.9)	41 (28.1)		—	—	
Education level			0.066			
Never attended school	96 (39.4)	87 (60.6)				
Less than primary school	131 (54.2)	121 (45.8)				
Primary school completed	68 (36.1)	93 (63.9)				
Secondary school completed	48 (58.1)	42 (41.9)				
High school completed	22 (53.2)	22 (46.8)				
College/pre-university/university completed	16 (74.1)	5 (25.9)				
Having insurance entitlement			**0.001**			
Insured	54 (28.2)	96 (71.8)		2.1	1.5, 3.2	**<0.001**
Not insured	327 (53.1)	274 (46.9)		—	—	

Note: 1n (unweighted) %(weighted); Median (Q1, Q3).

aOR, adjusted OR.

## Discussion

This study indicates that the usage of public healthcare services remains low overall, and there is a large disparity between the use of outpatient and inpatient public healthcare services. Factors such as sex, age, wealth quintile and health insurance entitlement were significantly associated with the usage of public healthcare for outpatient and/or inpatient services. Despite a low use of public healthcare, the study revealed that socioeconomically disadvantaged individuals tend to use the public sector more than others. However, it may not necessarily mean it excels in equity. Equity in healthcare means everyone has a fair opportunity to attain their full health potential, and no one is disadvantaged in achieving this potential. ^32^ To assess equity, we need to look beyond utilisation and consider factors like quality of care, health outcomes and accessibility, which were not measured in our study.

We observed a low proportion of public outpatient service utilisation (9.0%). Comparing our results with other studies in Cambodia and other settings is challenging because of the different target populations and contexts. However, these findings are lower than those of previous studies in Cambodia, in 2020 with an observed rate of 22% among individuals aged ≥40 years and in 2022 where a rate of 22.7% of overall public healthcare utilisation (combined outpatient and inpatient visits) was estimated among all age groups from the Cambodian Health and Demographic Survey (CDHS).^25 26^ Our results show a higher rate (79.9%) of private healthcare utilisation is similar to that observed in India, Nigeria and Nepal, where the government contribution to national health expenditure is low (<30%). The percentage of private-sector outpatient visits in India was 75%, 82% in Nigeria and 65% in Nepal. People in these settings may rely on private-sector healthcare services, which may be low cost and low quality.^33^ The findings suggest that public-sector outpatient services have not yet met users’ expectations and that the private sector is vital in providing outpatient care in Cambodia.

For inpatient care, a larger proportion of users (49.2%) reported public healthcare use in the 2023 WHS+ Cambodia, compared with a 2016 study reporting public inpatient care use in the last 12 months by 47.3% of respondents.^7^ This may be explained by the higher private inpatient care costs, users’ perception of cheaper public healthcare services and more recognition of public inpatient services in Cambodia. Public hospitals often have specialised facilities and personnel for handling complex cases and emergencies, making them preferred for inpatient care. It may also reflect the Ministry of Health’s efforts to improve public-sector services over the last several years and the organisation of insurance benefits offered at public health facilities. Another potential factor is that the private sector primarily focuses on outpatient services. For example, there are over 10 000 facilities designated for outpatient care, while options for inpatient services are much more limited. As a result, patients have fewer choices regarding inpatient care.

The higher proportion of women using public healthcare for outpatient services than men can be explained by their utilisation of public maternal health-related services, such as ANC, delivery, postnatal care and family planning at public healthcare facilities by women and their partners. According to the 2014 CDHS report, approximately 90% of pregnant women used ANC services at public healthcare facilities.^26^ These services also provide opportunities for women to receive other services, such as screening for diabetes, hypertension, HIV, TB and other conditions/diseases. This pattern may have been impacted by integrating traditional birth attendants and introducing the government’s midwifery incentive scheme in 2007.^34^

Furthermore, public services are used more often by individuals from lower socioeconomic levels than by those from higher socioeconomic levels, as previously observed in Cambodia and India.^23 25^ This is most likely due to the affordability of public-sector services. It is worth noting that most people with low economic status in Cambodia still rely on private outpatient (86.4%) and inpatient services (38.3%). Since they must spend OOP for private services, it partially contributes to the Cambodian high catastrophic health expenditure rate. In 2019, 17.7% of Cambodian households experienced catastrophic health expenditures at 10% threshold of their total household consumption expenditure.^35^ Additionally, 3.9% of households fell into poverty due to OOPE.^35^ However, our findings are inconclusive in understanding why individuals with limited financial resources choose private services. This could be due to the unavailability of necessary services or the fact that existing public-sector services fail to meet their expectations, suggesting further qualitative research to understand this phenomenon better and address this knowledge gap.

Our study estimated that 15.3% of the target population was covered by any social protection scheme. This is likely an underestimate, as our survey excluded collective households (eg, dormitories, apartments, barracks, rental rooms), which house many individuals with formal employment likely covered by the NSSF. This exclusion is significant because national data indicates that about 40% of the population is covered by HEF and/or NSSF.^20 21^

Despite this potential underestimation, our findings show that individuals with insurance entitlements use public healthcare more frequently than those without, as both the HEF and NSSF exclusively cooperate with public healthcare facilities.^25 36^

The results of our study are not surprising and confirm earlier evidence of the utilisation of public or private healthcare in Cambodia.^7^ Our findings indicate that private healthcare providers handle most outpatient visits in Cambodia, whereas public healthcare facilities are responsible for more than half of inpatient services and a few well-funded such as maternal and child care, and complex services such as depression or anxiety, and cancer. The low use of outpatient services may be due to the supply-side organisation targeting preventive care.^6 7^ Given this information, the Cambodian government should review its priorities and improvements to the public-sector services at the primary healthcare level, expanding the insurance scheme and mechanisms to engage with private healthcare providers. A strategy that works across both sectors, along with high insurance coverage, will push progress towards UHC.

However, this is easier said than done, as there is no perfect model for engaging and encouraging private healthcare providers in lower- to middle-income countries such as Cambodia.^37^ Nevertheless, we have some suggestions on how to approach this issue. First, the government should increase funding to the public sector to improve services and prioritise developments to ensure the public sector becomes a first-choice employer for the health workforce. Second, the government can develop and implement robust accreditation schemes for private and public healthcare providers. Accreditation of both public and private healthcare facilities can be done by the Ministry of Health and the government’s insurance schemes, as has been done not only in high-income countries but also in other low- and middle-income countries.^38^ Further steps can be taken, such as contracting high-performing private providers under a national insurance scheme, which can positively impact user choice and provider practice. Third, technology can be used to share the latest guidelines and regulations with private healthcare providers to ensure they have access to the most up-to-date guidelines or policies. Finally, as part of accreditation requirements, it should become mandatory for all private hospitals, clinics and cabinets to use the Ministry of Health’s Health Information System to manage patient electronic medical records, which can provide the government with more reliable information for designing effective policies. The long-term strengthening of regulations would be necessary to reduce unnecessary services, which can contribute to financial burdens for users.

To ensure that individuals from low- and middle-income households have access to quality healthcare services, social health insurance coverage should be expanded alongside improvements in public healthcare facilities to cater to the expected increase in public facility visits as part of the NSSF’s move towards universal insurance coverage in the country. The future vision is that most healthcare services will be provided at public facilities, and private providers will provide services that may not be widely available at public facilities. Improving primary healthcare as the preferred initial choice may significantly reduce secondary and tertiary care utilisation, making it of utmost importance. The success of such a health insurance scheme and healthcare care model has helped Thailand achieve UHC.^39 40^ However, this success could not have been achieved without the government’s increasing investment in improving public healthcare. This may be challenging for Cambodia, but it is still a model that Cambodia should aim for.

### Strengths and limitations

Our study has notable strengths that contribute to its validity. First, the sample had a representative design. We drew samples from all provinces and the capital, considering both urban and rural settings. Second, data collection was carefully planned and executed, with sufficient training and close monitoring. The limitations of our study include a non-response rate of approximately 15%, primarily because of the absence of eligible selected individuals in the selected household. However, this rate remains below the anticipated non-response rate for the survey of 20%, which was included in the sample size calculation. Second, we did not know whether people who used the pharmacy for outpatient services had received a prescription from a healthcare professional, which may have led to an overestimation of private healthcare use. However, to the best of our knowledge, individuals in Cambodia buy medicines without prescriptions from pharmacies.^41 42^ Third, individuals may be less likely to report sensitive healthcare visits, such as those related to HIV infection, provided at public healthcare facilities, leading to an underestimation of public healthcare. Fourth, in our study, participants were asked to recall their healthcare utilisation over the past year. This lengthy period may lead to underreporting of minor outpatient visits, as individuals might not remember specific instances of care. Consequently, the data could underestimate the frequency of such care. Finally, our study did not assess the quality of services, out-of-pocket expenses for public and private visits or treatment outcomes; therefore, our comparisons of the preference between public and private services did not incorporate quality aspects, which can be a significant factor.

## Conclusion

Our study confirms existing knowledge that private healthcare dominates outpatient services in Cambodia. However, public healthcare for inpatient services plays a more significant role than private healthcare services. Higher rates of public healthcare utilisation for outpatient and inpatient services were observed among individuals with low socioeconomic status and those with insurance. In contrast, women were more likely to use public outpatient care. In the Cambodian context, revisiting priority services and improving the quality of public healthcare at the primary healthcare level, particularly in rural and remote areas, expanding the coverage of services and social health protection and developing strategies to engage the private healthcare sector is crucial to progress towards UHC.

## Supplementary material

10.1136/bmjph-2024-001416 online supplemental file 1

10.1136/bmjph-2024-001416 online supplemental figure 1

10.1136/bmjph-2024-001416 online supplemental figure 2

## Data Availability

Data are available upon reasonable request.
