# Supplementary material for: Patterns and factors associated with healthcare utilisation in Cambodia: a cross-sectional study based on the World Health Survey Plus 2023
Source: BMJ Public Health. 2025 Feb 11;3(1):e001416. doi: 10.1136/bmjph-2024-001416 (PMC11883872; doi:10.1136/bmjph-2024-001416)
Supplement: online supplemental file 1 [file bmjph-3-1-s001.docx]

# Annexure 1. Principal Component Analysis

We categorized individuals into five socio-economic groups (poorest, poor, middle, rich, and richest) using wealth quintiles derived from principal component analysis (PCA). Household heads or representatives provided information on housing characteristics, sanitation facilities (e.g., toilet type, drinking water source), and household assets. Details of these variables are presented in the tables below. Binary response questions (yes/no) were coded as 1 for "yes" and 0 for "no". Questions with multiple response options were recoded into binary categories (presence/absence) in a similar manner. Continuous variables, such as the number of rooms, were retained in their original form.

To construct the SES index, we performed PCA separately for rural and urban settings using Stata's PCA function. The first principal component score was then used to rank individuals and assign them to wealth quintiles. Lower scores corresponded to lower SES, and higher scores indicated higher SES.

**Housing**

| Q0501 | Is this dwelling where you live…?  *INTERVIEWER: read options to the respondent.* | 1. Owned by the household head and fully paid off 2. Owned by the household head but not yet fully paid off 3. Owned by someone else in household and fully paid off 4. Owned by someone else in household but not yet fully paid off 5. Rented 6. Provided free of charge 7. 7 Other, specify: |  |
| --- | --- | --- | --- |
| Q0502 | How many rooms does this dwelling have in total, without counting the bathrooms or hallways? |  |  |

**environmental risk factors**

| Q0503 | What type of floor does your dwelling have? | 1. Hard Floor (Tile, Cement, Brick, Wood) 2. Earth Floor |  |
| --- | --- | --- | --- |
| Q0504 | What type of (exterior) walls does your dwelling have?  *(Circle main type)* | 1. Cement, Brick, Stone or wood 2. Mud/ Mud brick 3. Thatch and other 4. Plastic Sheet 5. Metal Sheet   7 Other, specify |  |

**Drinking water**

| Q0505 | What is the main source of drinking water for members of your household? | 1. Piped into dwelling 2. Piped into compound, yard or plot 3. Piped to neighbour 4. Public tap / standpipe 5. Borehole or tubewell 6. Protected dug well 7. Unprotected dug well 8. Protected spring 9. Unprotected spring 10. Rainwater collection 11. Tanker-truck 12. Cart with small tank / drum 13. Water kiosk 14. Bottled water 15. Sachet water 16. Surface water (river, stream, dam, lake, pond, canal, irrigation channel)   87 Other, specify | **1**.-> Go to Q0511  2.-> Go to Q0511  3.-> Go to Q0508  4.-> Go to Q0508  5.-> Go to Q0507  6.-> Go to Q0507  7.-> Go to Q0507  8.-> Go to Q0507  9.-> Go to Q0507  10-> Go to Q0507  11.-> Go to Q0508  12.-> Go to Q0508  13.-> Go to Q0508  14.-> Go to Q0506  15.-> Go to Q0506  16.-> Go to Q0508  87-> Go to Q0507 |
| --- | --- | --- | --- |
| Q0506 | What is the main source of water used by members of your household for other purposes, such as cooking and hand washing? | 1. Piped into dwelling 2. Piped into compound, yard or plot 3. Piped to neighbour 4. Public tap / standpipe 5. Borehole or tubewell 6. Protected dug well 7. Unprotected dug well 8. Protected spring 9. Unprotected spring 10. Rainwater collection 11. Tanker-truck 12. Cart with small tank / drum 13. Water kiosk 14. Bottled water 15. Sachet water 16. Surface water (river, stream, dam, lake, pond, canal, irrigation channel)   87 Other, specify | 1.-> Go to Q0511  2.-> Go to Q0511  11-> Go to Q0508  12-> Go to Q0508  13-> Go to Q0508  14-> Go to Q0511  15-> Go to Q0511  16-> Go to Q0508 |

**Sanitation**

| Q0514 | What kind of toilet facility do members of your household usually use?  [If flush or pour flush probe where does it flush to]  [If not possible to determine, ask permission to observe facility] | 1. Flush 2. Flush/pour flush to piped sewer system 3. Flush/pour flush to septic tank 4. Flush/pour flush to pit latrine 5. Flush/pour flush to open drain 6. Flush/pour flush to don’t know where 7. Pit latrine with slab 8. Pit latrine without slab / Open pit 9. Twin pit with slab 10. Twin pit without slab 11. Other composting toilet 12. Bucket 13. Container based sanitation 14. Hanging toilet / hanging latrine 15. No facility / Bush / Field   87 Other (specify) | 15-> Go to Q0519 |
| --- | --- | --- | --- |

Section 0700: Assets

| Q0701 | A television? | 1. yes 2. no |
| --- | --- | --- |
| Q0702 | A motorcycle or motor scooter? | 1. yes 2. no |
| Q0703 | A car or truck? | 1. yes 2. no |
| Q0704 | Electricity? | 1. yes 2. no |
| Q0705 | A bicycle? | 1. yes 2. no |
| Q0706 | A microwave oven? | 1. yes 2. no |
| Q0707 | Hot running water? | 1. yes 2. no |
| Q0708 | A washing machine? | 1. yes 2. no |
| Q0709 | A dishwasher? | 1. yes 2. no |
| Q0710 | A refrigerator? | 1. yes 2. no |
| Q0711 | A fixed-line telephone? | 1. yes 2. no |
| Q0712 | A mobile / cellular telephone? | 1. yes 2. no |
| Q0713 | A VCR (video) or DVD player? | 1. yes 2. no |
| Q0714 | A computer? | 1. yes 2. no |
| Q0715 | A radio? | 1. yes 2. no |
| Q0716 | Livestock (cattle, goats, pigs, poultry)? | 1. yes 2. no |
| Q0717 | Internet access in the home? | 1. yes 2. no |
| Q0718 | An air-condition (cooling) system in the home? | 1. yes 2. no |
| Q0719 | A heating system in the home? | 1. yes 2. no |
| Q0720 | Does your household have one or more domestic servants? | 1. yes 2. no |
| Q0721 | Does any member of this household own any agricultural land? | 1. yes 2. no |
| Q0722 | Does any member of this household own a dwelling (other than this dwelling where you live)? | 1. yes 2. no |

I would now like to know if you own any land – and the approximate value (cash equivalent amount). I know this is sensitive information and will not share this with any persons outside of the survey team.

|  | | **A**.  *If Yes, 🡺 Column B*  *If no 🡺 skip to Q0724* | **B.**  About how much is this worth in total? *(cash equivalent)* |
| --- | --- | --- | --- |
| Q0723 | Do you own any land or property? | 1. Yes 2. No 🡺 b   8 *DK* | *-8*  *DK*  *-97 Refused* |
