# Supplementary figures and images for: Patterns and factors associated with healthcare utilisation in Cambodia: a cross-sectional study based on the World Health Survey Plus 2023
Source: BMJ Public Health. 2025 Feb 11;3(1):e001416. doi: 10.1136/bmjph-2024-001416 (PMC11883872; doi:10.1136/bmjph-2024-001416)

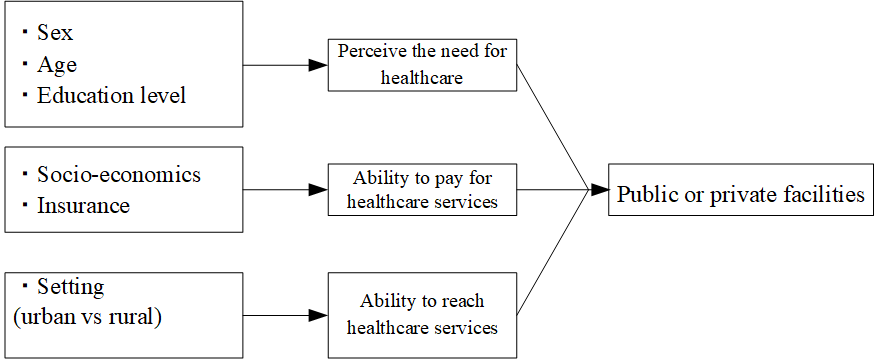

Supplement: online supplemental figure 1 [file bmjph-3-1-s002.png]

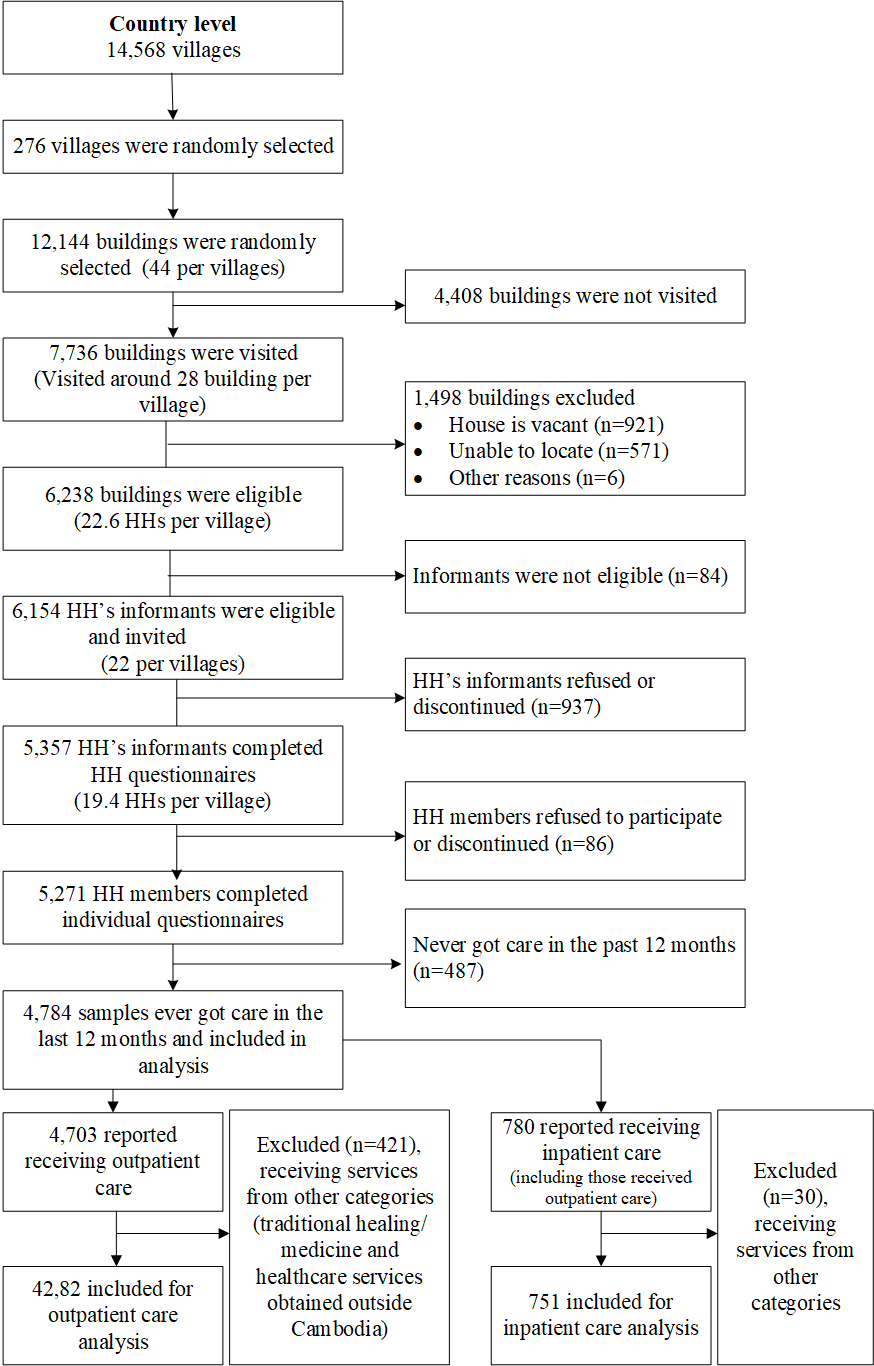

Supplement: online supplemental figure 2 [file bmjph-3-1-s003.png]
